# Non-Random Chromosome Segregation in Stem Cells

**DOI:** 10.1371/journal.pbio.0050125

**Published:** 2007-04-17

**Authors:** Richard Robinson

There are many small bits of conventional wisdom in biology whose general truths may be widely assumed, despite limited evidence from rather narrow circumstances. One such bit of wisdom concerns the random separation of chromosomes into daughter cells during mitosis. In a new study, Thomas Rando and colleagues show that for muscle stem cells, that separation is anything but random.

Before every mitotic cell division, each chromosome must be copied in full to ensure that each daughter cell has a complete complement of genes as it begins its new life. During replication, the double helix is pulled apart, and each strand serves as the template for creating a new opposite strand. After replication, the two copies, called sister chromatids, are each half old and half brand new (hence the name “semi-conservative” replication). During mitosis, these chromatid pairs are separated from each other, one pulled into one nascent daughter cell, the other pulled into the other. If we were to paint both old strands white and both new strands green, say, and watch one complete cycle of replication and mitosis, we would first see the two original white strands pulled apart, then make new green partners, and finally be pulled into daughter cells, one green-white pair going one way, the other going the other.

Each human cell contains 46 chromosomes, and during mitosis, each daughter cell receives 46 chromatids (which are rechristened as chromosomes as soon as they separate from their sister). If we were to paint all 92 old strands white and 92 new strands green and watch them during the first round of cell division, we’d first see 92 older white strands get pulled apart, then they would make 92 green partners, and then we would see 92 green-white pairs get pulled into daughter cells, 46 going one way, 46 going the other.

But if we went on to watch one of these cells go through the next round, it would not be so easy to predict what would happen to our white strands. We begin this round with only 46 older white strands, one in each chromosome, and after replication, each four-stranded chromatid pair would contain only one white strand. During mitosis, each chromatid pair is separated, and 46 white strands would get pulled into daughter cells. But what determines how these 46 are distributed? Is it random? Half to each daughter? Or are all 46 older white strands pulled into the same daughter cell?

This is the question Rando and colleagues set out to ask about stem cells in muscle tissue. Like other stem cells, muscle stem cells undergo repeated rounds of DNA replication and cell division. During most divisions, one daughter retains the stem cell identity and function, while the other differentiates and matures into a muscle cell. Inevitably, mutations are introduced during replication, and after many cycles, such new mutations can become fatal to the cell bearing them. Based on this fact, the authors hypothesized that stem cells might be more likely than other cells to direct the older, more pristine, DNA strands into the daughter cell that remained a stem cell.

To test this hypothesis, they exposed chromosomes to modified nucleotides, containing either chlorine, bromine, or iodine (called CldU, BrdU, and IdU). During replication, these become incorporated into the newly synthesized strand, and can be visualized with a set of antibodies. Because it fits well into the replicated DNA, BrdU has long been used to stain chromosomes. Chlorine is a little smaller than bromine, and iodine a little larger, but these too do a serviceable job and, in small doses, don’t disrupt the replication process.

**Figure:**
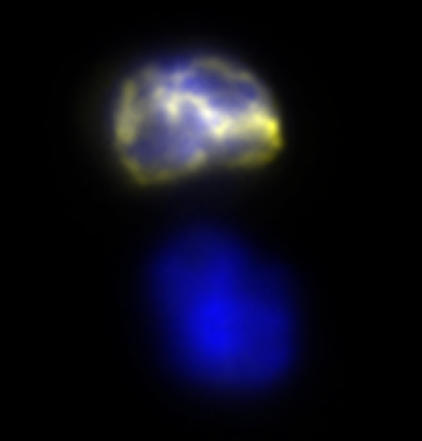
When stem cells divide, the sister chromatids containing the older template DNA strands (yellow) may all segregate to one daughter cell, reflecting the different fates of the progeny of that cell division.

The authors began by injuring muscle to induce the resident stem cells to divide, exposing them first to CldU, in effect painting the new strands red. Twelve hours and one cell cycle later, they exposed the cell to IdU (green), and allowed them to divide once more. If chromatid separation was truly random, then after these two cell divisions, the red label would be distributed equally among the second-division (green-labeled) daughter cells (the older, “white” DNA remained unlabeled). Instead, they found that nearly half the cells displayed either an excess or deficiency of red label, indicating that the newer and older template strands were not distributed randomly; instead, they were segregated by age into one or the other daughter.

Was there a relationship between the age of the DNA and the fate of the cell containing it? To find out, the authors isolated pairs of cells derived from a single stem cell, and compared the proportion of newer DNA (this time marked with BrdU) with the expression of a marker for muscle differentiation, Desmin. They found that cells with the newer template strands had the most Desmin; this, more error-prone DNA correlated with differentiation, while older, more pristine DNA was retained in the new stem cell. Conversely, in most of those pairs with an equal division of older DNA, Desmin expression was also equally expressed. The propensity to allocate older DNA asymmetrically was lost as cells became more differentiated, because asymmetrical allocation did not occur in the vast majority of dividing myoblasts, which are descendants of stem cells but fully committed to becoming mature muscle.

The theoretical possibility of strand segregation during stem cell self-renewal has long been recognized and has been termed the “immortal strand hypothesis.” But little evidence for it has been found until now, and certainly not repeated strand segregation through multiple mitoses as stem cells undergo proliferative expansion. Instead, reports of limited counterexamples, plus the appealingly simple notion of random separation, have led to the conventional wisdom that chromatids always separate randomly. These new experiments overturn that conventional wisdom, at least for muscle stem cells. It seems plausible that similar phenomena will be found in other stem cells, now that there are the tools and the added incentive to look.

This discovery is more than a reminder that some of the things we think we know “just ain’t so.” Measurements of stem cell proliferation have typically relied on measuring the dilution of labels such as BrdU over time, on the assumption that it is equally distributed into all daughters. Those results will have to be reconsidered. Slowly dividing cells have often been identified in tissues by their long retention of labels, which now seems questionable.

The results of this study raise many interesting questions, including the nature of the segregation mechanism; whether the daughter cell identity is determined by chromosome segregation, or vice versa; and how rigorous the segregation is within any pair of daughter cells (the data presented do not answer whether all 46 older chromosomes, or just some large fraction of them, end up together). These questions must be explored in future work.

